# Current Hospital Topics

**Published:** 1907-03-02

**Authors:** 


### HOSPITAL ADMINISTRATION.

### CONSTRUCTION AND ECONOMICS.

### CURRENT HOSPITAL TOPICS.

#### The Evelina Hospital Secretaryship.

There is a vacancy in the office of secretary to this hospital, the salary of which is £250 per annum. Applications with testimonials must be sent in to the hospital not later than to-day, Saturday, March 2, 1907. We have ascertained that the election is an open one, and that the appointment will go to the best man. The requirements are a good knowledge and experience of hospital administration, sufficient character and administrative ability on the part of the secretary to enable him to occupy a position of independence, whilst maintaining the most cordial relations with the rest of the staff; ability to raise funds; and a knowledge of the business side of hospital affairs so as to secure the maximum of economy with efficiency. We hope that the committee's desire to appoint the best available man will induce other hospitals to co-operate, by encouraging any gentleman known by them to be efficient, to present himself as a candidate for the vacant office. We venture to congratulate the committee of the Evelina Hospital on the wise example they have set by resolving to appoint no one who cannot produce evidence of previous training, an aptitude for hospital management, a good education, and the possession of those qualities which constitute a gentleman.

#### Hospital Sunday in the Provinces.

Some interesting figures have been prepared in support of the Hospital Sunday movement in Sunderland. It appears that the total expenditure upon 12,436 patients, of whom 2,745 were in-patients, treated at the Sunderland Infirmary last year was £11,227, towards which the working classes subscribed the handsome sum of £7,038. We are informed that nearly two-thirds of the total expenditure was contributed by the working classes. This is a striking fact, which is greatly to the credit of the working men of Sunderland. It appears to have failed, however, to stimulate the middle-class inhabitants of Sunderland to do their duty by supporting the Sunderland Infirmary. This view is confirmed by the circumstance that Sunderland, with a population of 146,000, according to the last census, only contributed £535 through the Hospital Sunday Fund. Norwich, with a population of only 112,000, subscribed £2,002. Derby, with 106,000 population, subscribed £1,332; and Southampton, with 105,000 population, subscribed £1,130 to the hospitals through the Sunday Fund. It is clear from these figures that, for some reason, for which we are unable to account, the classes, who chiefly support the voluntary hospitals in most parts of the country, do not in Sunderland appear to be alive to the importance and privilege of rendering a measure of personal service in the days of health in the cause of the sick. We hope that the energetic action of the *Sunderland Echo* and the infirmary authorities may lead to a development of the Hospital Sunday movement in Sunderland, and that the well-to-do members of this important community may be aroused to take their share in the work of ministering to the least fortunate members who, from no fault of their own, may be overtaken by accident or attacked by disease. We regret to notice that in another provincial town—Leicester—the Hospital Sunday collections last year fell off from £2,202 to £1,982, whereas the contributions from the Hospital Saturday Fund amounted to £10,863, an increase of £600 compared with the previous year. Leicester is a very prosperous town; it is the centre of the shoe trade, and we venture to hope that the Hospital Sunday collections during 1907 will better represent the wealth and importance of this great manufacturing centre.

#### Central Hospital Council for London.

This Central Hospital Council consists of the representatives of fifteen general and five special hospitals in London. Its members collectively number thirty-three, of whom fourteen are members of the medical profession. Each of the large hospitals with a medical school appoints two representatives, with a third in the case of St. Thomas's Hospital, who is associated with the Nightingale School. For several years this Council was comparatively inactive; recently it has taken up two matters which mark a new departure. They are causing some discussion in hospital circles at the present time because they relate to subjects involving legislative action, and to proceedings in regard to the incorporation of a body, the well-being of which is vital to the best interests of all the voluntary hospitals. Some hospitals, we understand, hold the opinion that any representative they may send to the Central Hospital Council can have, and ought to have, no power, without reference back, to commit his institution to a policy involving legislative action, or to take any step which may cause annoyance or give offence to important officers or supporters of a particular institution or institutions. Underlying this, there is the further principle that no representative of any voluntary hospital should be endowed with powers to support any policy which might entail expenditure, without first obtaining the consent and approval of those whom he represents. There can be no doubt that if this Council is to continue and to prove of practical value, it is essential that its proceedings shall be governed by bye-laws which have received the general approval of the governing authorities of each of the hospitals represented. It is manifestly *ultra vires* for a Council of this kind to promote a Bill in Parliament, for example, without having first submitted that Bill to each of the hospitals concerned. Such a proceeding not only commits every one of the hospitals represented on the Council to a course which they may not approve, at least in form, but it may also involve them in liability, which any hospital committee might consider it undesirable to incur. They might hold, for instance, that hospital funds were not available for the purpose of promoting general legislation, or to pay the expenses of opposition to measures brought before Parliament. A Council of this kind cannot properly speak in the name of the hospitals, where matters of principle or matters of controversy are involved, unless the authority of each hospital represented, to the course of action which the majority of its members may consider it desirable to take, has been obtained. Unless, therefore, they already exist, no time should be lost in formulating by-laws, which will bring the Council and its proceedings into harmony with the feelings and views of the governing bodies, whose representatives constitute the members of the Council.

